# Fluoroquinolones and the Risk for Methicillin-resistant *Staphylococcus aureus* in Hospitalized Patients^1^

**DOI:** 10.3201/eid0911.030284

**Published:** 2003-11

**Authors:** Stephen G. Weber, Howard S. Gold, David C. Hooper, A.W. Karchmer, Yehuda Carmeli

**Affiliations:** * Beth Israel Deaconess Medical Center and Harvard Medical School, Boston, Massachusetts, USA; † Massachusetts General Hospital and Harvard Medical School, Boston, Massachusetts, USA

## Abstract

To determine whether fluoroquinolone exposure is a risk factor for the isolation of *Staphylococcus* *aureus* and whether the effect is different for methicillin-resistant *S.* *aureus* (MRSA) versus methicillin-susceptible *S.* *aureus* (MSSA), we studied two case groups. The first case group included 222 patients with nosocomially acquired MRSA. The second case group included 163 patients with nosocomially acquired MSSA. A total of 343 patients admitted concurrently served as controls. Outcome measures were the adjusted odds ratio (OR) for isolation of MRSA and MSSA after fluoroquinolone exposure. Exposure to both levofloxacin (OR 5.4; p < 0.0001) and ciprofloxacin (OR 2.2; p < 0.003) was associated with isolation of MRSA but not MSSA. After adjustment for multiple variables, both drugs remained risk factors for MRSA (levofloxacin OR 3.4; p < 0.0001; ciprofloxacin OR 2.5; p = 0.005) but not MSSA. Exposure to levofloxacin or ciprofloxacin is a significant risk factor for the isolation of MRSA, but not MSSA.

Methicillin-resistant *Staphylococcus aureus* (MRSA) has been implicated as a pathogen in hospital-acquired infections since the 1960s (*1*). During the 1990s, the proportion of nosocomial infections caused by MRSA increased substantially, and MRSA is now a leading cause of such infections in the United States (*2*). According to data from the SENTRY Antimicrobial Surveillance Program, approximately 40% of *S. aureus* isolates recovered in intensive care units (ICU) are resistant to methicillin (*3*). Recently, MRSA infections acquired in the community have been identified as emerging pathogens responsible for substantial disease and death (*4*,*5*). While no satisfactory explanation exists for the recent proliferation of MRSA, expanded use of antimicrobial drugs in sites outside the hospital has been suggested as a major contributor to emerging resistance in the community (*6*).

Fluoroquinolones are among the most commonly prescribed classes of antimicrobial drugs in both the hospital and in the community. Ciprofloxacin, one of the first fluoroquinolones to gain extensive clinical use, was originally heralded for its activity against a broad range of pathogens, including MRSA (*7*). However, by the early 1990s, many MRSA isolates from clinical specimens were found to be resistant to ciprofloxacin (*8*). The next generation of fluoroquinolones, including levofloxacin, was introduced during the second part of the 1990s and promised improved activity against gram-positive pathogens. Unfortunately, screening of large numbers of staphylococcal bloodstream isolates as part of the SENTRY Antimicrobial Surveillance Program demonstrated resistance to many of the newest fluoroquinolones as well (*9*).

Several recent investigations offer preliminary evidence that suggests that the fluoroquinolones themselves may actually predispose patients to infection with or carriage of MRSA. A comparison of microbiology laboratory data with antimicrobial reimbursement reports found a significant correlation between ciprofloxacin prescriptions and the isolation of MRSA (*10*). Two case-control studies examining risk factors for MRSA have found a significant association between fluoroquinolone exposure and MRSA isolation or infection (*11*,*12*). Preliminary analysis from one also suggested a difference in risk between members of the class. Additionally, a prospective study examining the impact of nasally administered mupirocin ointment on MRSA carriage also identified fluoroquinolone exposure as a risk factor for MRSA carriage (*13*). However, none of these studies was designed specifically to examine the risk associated with fluoroquinolones. Moreover, the design of the prior case-control investigations, as a result of the inappropriate use of patients colonized or infected with sensitive strains for controls, may have yielded biased results. Thus, the association between fluoroquinolone exposure and MRSA remains to be confirmed.

This study was specifically designed to determine whether exposure to fluoroquinolones is a risk factor for the subsequent isolation of *S. aureus*, and whether the effect is different for MRSA versus methicillin-susceptible *S. aureus* (MSSA). In addition, we sought to preliminarily explore any difference in risk between levofloxacin and ciprofloxacin.

## Methods

The study was performed at the Beth Israel Deaconess Medical Center, a 640-bed tertiary care teaching hospital in Boston, Massachusetts. The case-case-control method was used. As originally described by Kaye et al., this technique more rigorously upholds the epidemiologic standard that members of a control group be selected independently of their exposure status (*14*,*15*). A number of subsequent studies and commentary have upheld the utility of this method as providing the most accurate estimates of risk in studies of antimicrobial drug resistance (*15*–*21*). The case-case-control design is actually composed of two parallel case-control studies. Here, the first group of cases consisted of patients from whom nosocomially acquired MRSA was isolated. The second case group was comprised of patients from whom nosocomially acquired MSSA was recovered. *S. aureus* isolates were identified by using standard laboratory procedures. Resistance was determined according to the National Committee for Clinical Laboratory Standards guidelines with automated microdilution testing with the VITEK 2 system (bioMérieux, Hazelwood, MO). In addition, oxacillin resistance was confirmed on MRSA screening agar. A microbiology laboratory database of clinical cultures was searched to identify patients from whom *S. aureus* was isolated from November 1, 1999, to August 1, 2001. Any patient from whom *S. aureus* was first recovered during the initial 72 hours after admission to the medical, surgical, or obstetric services was presumed to have acquired the organism before hospitalization and was excluded. The control group for each of the two component studies was composed of a computer-generated random sample of patients admitted during the same period to the medical, surgical, or obstetric services from whom *S. aureus* was not isolated. To be included, control patients needed to be hospitalized for at least 72 hours. The same group of control patients was used for both cases with MRSA and MSSA.

Data regarding candidate risk factors were collected from existing administrative, pharmacy, and laboratory databases by using a relational database management system (Access; Microsoft Corporation, Redmond, WA). In addition to patient sex and age, coexisting medical conditions were analyzed; these included the presence or absence of cardiovascular, lung, hepatic or renal disease; previous organ transplant; AIDS; malignancy; and diabetes mellitus. Factors specifically relating to hospitalization were collected and analyzed, including transfer from another hospital or care facility, prior surgical procedure or ICU stay, presence or absence of intravenous line, emergent admission, admission service (medicine, surgery, or obstetrics), and the number of days at risk for infection. For case-patients, the last was equal to the number of days of hospitalization before the first isolation of *S. aureus*. For control patients, the number of days at risk was defined as the total length of stay. While the primary objective was to measure the specific risk associated with exposure to levofloxacin or ciprofloxacin, also considered was exposure to a number of other antimicrobial agents, including vancomycin, penicillins, β-lactam/β-lactamase inhibitor combinations, first- and second-generation cephalosporins, third-generation cephalosporins, carbapenems, clindamycin, and metronidazole. During the study period, levofloxacin and ciprofloxacin were the only fluoroquinolones used routinely at the Beth Israel Deaconess Medical Center. To be included as an exposure, any antimicrobial drug dosing, surgical procedure, ICU stay, and placement of intravenous line had to occur before the isolation of *S. aureus* in case-patients.

According to the hospital’s infection-control policy, all patients from whom MRSA is isolated are placed on contact precautions in a private room. In addition, patients from whom resistant bacteria have been isolated during prior hospitalizations are automatically flagged and isolated at the time of readmission to minimize cross-contamination. Appropriate adherence to these policies by the staff is consistent across different care units. During the study period, patients diagnosed with MRSA infection were treated with vancomycin.

The SAS software package (SAS Institute, Cary, NC) was used for all statistical analysis. Because the opportunity for both exposures and outcome (i.e., a clinical culture positive for *S. aureus*) would necessarily increase with the number of days at risk, simple univariate analysis does not appropriately reflect the individual risk associated with each of the candidate risk factors. Therefore, a two-variable logistic regression model adjusted for time at risk was used to specify the individual risk associated with levofloxacin, ciprofloxacin, and each of the other candidate risk factors. Odds ratios (OR), 95% confidence intervals (CI), and p values were calculated for each.

To quantify more accurately the specific risk for *S. aureus* isolation after exposure to levofloxacin or ciprofloxacin, variables with a p value of <0.05 in the adjusted univariate analysis were included in a logistic regression model along with each of the fluoroquinolones. Separate models were constructed for MRSA and MSSA cases. Automated selection functions were not used. Candidate risk factors that reached statistical significance (p < 0.05) were retained in the multivariable model to adjust the risk associated with levofloxacin and ciprofloxacin. For each of the two case groups, candidate risk factors that did not reach statistical significance in the multivariable model were only allowed to remain in the model as confounders if their removal changed the coefficient for levofloxacin or ciprofloxacin by >10%. Interaction terms for each of the risk factors included in the final model were similarly included if these criteria were met. Each of the final multivariable models was tested for overfitting by using the bootstrap method (1,000 bootstrap samples of all of the data). Goodness-of-fit of the final models was evaluated with the Hosmer-Lemeshow test. The project was approved by the institutional review board of the Beth Israel Deaconess Medical Center

## Results

Two hundred twenty-two patients with nosocomial MRSA and 163 with nosocomial MSSA were identified and served as the two case groups. For both, 343 randomly selected inpatients hospitalized for at least 72 hours were identified as controls. The mean age of MRSA and MSSA cases was 66.2 and 63.3 years, respectively. Control patients, with a mean age of 57.6 years, were significantly younger. Compared to 39.9% of controls, 56.8% of MRSA patients and 55.8% of MSSA patients were men. The mean number of days in hospital before the first positive culture was 12.4 and 7.7 for MRSA and MSSA patients, respectively. In comparison, the number of days at risk for control patients was 6.7 days, significantly shorter than for those from whom resistant organisms were isolated. In total, 67.6% of the MRSA patients were exposed to one of the two fluoroquinolones under study. This value was significantly greater than the number for MSSA (22.7%) or control (21.0%) patients. Of all MRSA isolated, 97% were resistant to fluoroquinolones as opposed to 8% of MSSA strains. As described elsewhere, approximately 80% of isolates tested were variants of a single pulsed-field gel electrophoresis type (*22*). In addition, during the study, no epidemiologically related outbreak of *S. aureus* disease was detected at the institution.

### MRSA Versus Control Patients

The results of univariate analyses for MRSA adjusted for days at risk are shown in Table 1. Exposure to both levofloxacin (OR 5.4; p < 0.0001) and ciprofloxacin (OR 2.2; p = 0.0027) was more common among MRSA case-patients than controls. In addition, male patients were more likely to be case-patients than controls (OR = 1.8; p = 0.014). When compared with patients <50 years of age, those 50–75 years of age (OR = 2.4; p < 0.0001) and >75 years of age (OR = 2.9; p < 0.001) were more likely to be cases. Cardiovascular (OR 1.8; p = 0.0043), lung (OR 6.8; p < 0.0001), renal (OR 2.0; p = 0.0114), and hepatic (OR 2.4; p = 0.0107) disease were all more common among cases than controls. Hospital transfer (OR 2.9; p < 0.0001), ICU stay (OR 7.7; p < 0.0001), intravenous line (OR 2.1; p = 0.0008), and emergency room admission (OR 2.2; p < 0.0001) were also significantly more frequent among case-patients than controls. Patients admitted to the obstetrics service, when compared with those admitted to the medical service, were more likely to be controls than case-patients (OR 0.1; p < 0.0001). In addition to the fluoroquinolones, exposure to several other antimicrobial drugs was significantly more common among cases than controls, including vancomycin (OR 4.0; p < 0.0001), penicillin (OR 2.1; p = 0.0042), third-generation cephalosporins (OR 3.7; p < 0.0001), clindamycin (OR 5.4; p < 0.0001), and metronidazole (OR 4.2; p < 0.0001).

**Table 1:** Results of univariate analysis for MRSA cases^a^

Risk factor	Uncolonized (n = 343)		MRSA cases (n = 222)				
	no.	%	no.	%	OR (95% CI)	p value	
Demographic							
Male	137	39.9	126	56.8	1.84 (1.27 to 2.67)	0.001	
Age	mean = 57.6 y		mean = 66.2 y			<0.001	
<50	131	38.2	39	17.6	—	—	
51–75	137	39.9	110	49.5	2.39 (1.50 to 3.83)	<0.001	
>75	75	21.9	73	32.9	2.86 (1.70 to 4.79)	<0.001	
Coexisting condition							
Cardiovascular disease	186	54.2	156	70.3	1.77 (1.20 to 2.31)	0.004	
Lung disease	57	16.6	130	58.6	6.74 (4.43 to 10.26)	<0.001	
Renal disease	30	8.7	46	20.7	2.02 (1.17 to 3.47)	0.01	
Hepatic disease	17	5.0	28	12.6	2.43 (1.23 to 4.79)	0.01	
Organ transplant	4	1.2	2	0.9	0.52 (0.08 to 3.36)	0.49	
AIDS	5	1.5	4	1.8	1.25 (0.30 to 5.14)	0.76	
Malignancy	54	15.7	32	14.4	0.98 (0.59 to 1.64)	0.94	
Diabetes mellitus	67	19.5	56	25.2	1.26 (0.81 to 1.96)	0.31	
Hospital factors							
Transfer	32	9.3	53	23.9	2.88 (1.71 to 4.83)	<0.001	
Surgical procedure	148	43.1	96	43.2	0.74 (0.51 to 1.09)	0.12	
ICU stay	54	15.7	144	64.9	7.66 (5.01 to 11.71)	<0.001	
Intravenous line	59	17.2	80	36.0	2.06 (1.38 to 3.16)	<0.001	
Emergent admission	149	43.4	136	61.3	2.20 (1.51 to 3.21)	<0.001	
Admission service							
Medical	174	50.7	154	69.4	—	—	
Obstetrical	62	18.1	2	0.9	0.05 (0.01 to 0.21)	<0.001	
Surgical	107	31.2	66	29.7	0.74 (0.49 to 1.10)	0.14	
Antimicrobial drugs							
Any fluoroquinolone	72	21.0	150	67.6	5.41 (3.60 to 8.11)	<0.001	
Levofloxacin	42	12.2	109	49.1	5.36 (3.45 to 8.32)	<0.001	
Ciprofloxacin	34	9.9	62	27.9	2.16 (1.31 to 3.56)	0.002	
Vancomycin	36	10.5	94	42.3	3.98 (2.49 to 6.34)	<0.001	
Penicillin	36	10.5	56	25.2	2.08 (1.26 to 3.45)	0.004	
β-lactam and inhibitor	5	1.5	13	5.9	2.74 (0.88 to 8.46)	0.08	
First-generation cephalosporin	77	22.4	45	20.3	0.77 (0.49 to 1.21)	0.25	
Third-generation cephalosporin	28	8.2	74	33.3	3.66 (2.18 to 6.13)	<0.001	
Carbapenem	3	0.9	7	3.2	1.63 (0.37 to 7.10)	0.52	
Clindamycin	9	2.6	32	14.4	5.36 (2.39 to 12.01)	<0.001	
Metronidazole	42	12.2	104	46.8	4.20 (2.70 to 6.55)	<0.001	

^a^All results adjusted for time at risk. MRSA, methicillin-resistant *Staphylococcus aureus*; OR, odds ratio; CI, confidence interval; ICU, intensive care unit.

Multivariable models to quantify the specific risk associated with levofloxacin or ciprofloxacin were constructed for MRSA cases. The results are shown in Table 2. After adjustment for other significant and confounding variables, including other antibiotic exposures, exposure to either levofloxacin (OR 3.4; p = 0.0001) or ciprofloxacin (OR 2.5; p = 0.0049) was more common among MRSA cases than controls. No interaction terms met the significance criteria for inclusion in the final model.

**Table 2:** Results of multivariable analysis^a^

Risk factor	MRSA cases		MSSA cases		
	OR (95% CI)	p value	OR (95% CI)	p value	
Primary covariates					
Levofloxacin	3.38 (1.94 to 5.90)	<0.001	0.69 (0.34 to 1.40)	0.30	
Ciprofloxacin	2.48 (1.32 to 4.67)	0.005	0.47 (0.21 to 1.02)	0.06	
Other covariates					
Lung disease	3.94 (2.43to6.40)	<0.001	2.33 (1.43 to 3.81)	<0.001	
Renal disease	*		1.98 (1.03 to 3.80)	0.04	
Penicillin	*		1.78 (0.93 to 3.39)	0.08	
Metronidazole	1.92 (1.10 to 3.37)	0.02	1.29 (0.65 to 2.56)	0.46	
ICU stay	5.33 (3.28 to 8.68)	<0.001	4.60 (2.90 to 7.30)	<0.001	
Emergent admission	1.74 (1.09 to 2.78)	0.02	1.90 (1.17 to 3.08)	0.01	
Admission service					
Medical	*		—	—	
Obstetrical	*		0.29 (0.08 to 1.05)	0.06	
Surgical	*		1.82 (1.12 to 2.97)	0.02	

^a^All results adjusted for time at risk. Only variables significant (p < 0.05) on univariate analysis were included in final models. Several variables (indicated with an asterisk) met criteria for inclusion in the MSSA (methicillin-susceptible *Staphylococcus aureus*), but not the MRSA (methicillin-resistant *Staphylococcus aureus*) model. OR, odds ratio; CI, confidence interval; ICU, intensive care unit.

### MSSA Versus Control Patients

The results of univariate analyses adjusted for days at risk for MSSA are shown in Table 3. As opposed to the situation for those with MRSA, MSSA cases were no more likely than controls to have been exposed to levofloxacin or ciprofloxacin. MSSA case-patients were more likely than controls to be male (OR 1.9; p = 0.0011). Patients >75 years old were more likely than those <50 to be case-patients (OR 2.1; p = 0.0037). Some coexisting conditions were more common among case-patients than controls, including cardiovascular (OR 2.1; p = 0.0004), lung (OR 3.0; p < 0.0001), renal (OR 2.1; p = 0.0119), and hepatic disease (OR 3.2; p = 0.0006). Hospital transfer (OR 2.1; p = 0.0076), ICU stay (OR 6.4; p < 0.0001), intravenous line (OR 2.0; p = 0.0023), and emergency room admission (OR 2.0; p = 0.0009) were also more likely among case-patients than controls. When compared with patients admitted to the medical service, those admitted to the surgical service were more likely to be cases (OR 1.5; p = 0.0497), and those admitted to obstetrics were more likely to be controls (OR 0.1; p = 0.0002). Among antimicrobial agents, only exposure to penicillin (OR 2.2; p = 0.0039) and metronidazole (OR 1.7; p = 0.043) was more frequent among cases than controls.

**Table 3:** Results of univariate analysis for MSSA cases^a^

Risk factor	MSSA cases (n = 163)			
Demographic	No.	%	OR (95% CI)	p value
Male	91	55.8	1.89 (1.29 to 2.77)	0.001
Age	Mean = 63.3 y.			
<50	42	25.8	—	—
51–75	68	41.7	1.50 (0.95 to 2.37)	0.08
>75	53	32.5	2.10 (1.27 to 3.45)	0.004
Coexisting condition				
Cardiovascular disease	117	71.8	2.08 (1.39 to 3.13)	<0.001
Lung disease	61	37.4	2.95 (1.92 to 4.54)	<0.001
Renal disease	28	17.2	2.06 (1.17 to 3.62)	0.01
Hepatic disease	24	14.7	3.16 (1.64 to 6.10)	<0.001
Organ transplant	2	1.2	1.00 (0.18 to 5.60)	0.99
AIDS	1	0.6	0.39 (0.00 to 3.42)	0.40
Malignancy	30	18.4	1.21 (0.74 to 1.98)	0.46
Diabetes mellitus	38	23.3	1.25 (0.80 to 1.98)	0.33
Hospital factors				
Transfer	29	17.8	2.11 (1.22 to 3.64)	0.01
Surgical procedure	62	38.0	0.78 (0.52 to 1.15)	0.20
ICU stay	89	54.6	6.38 (4.14 to 9.83)	<0.001
Intravenous line	50	30.7	1.99 (1.28 to 3.09)	0.002
Emergent admission	97	59.5	1.90 (1.30 to 2.79)	<0.001
Admission service				
Medical	85	52.1	—	—
Obstetrical	3	1.8	0.10 (0.03 to 0.34)	<0.001
Surgical	75	46.0	1.49 (1.00 to 2.21)	0.05
Antimicrobial drugs				
Any fluoroquinolone	37	22.7	0.96 (0.60 to 1.53)	0.86
Levofloxacin	24	14.7	1.09 (0.62 to 1.90)	0.77
Ciprofloxacin	14	8.6	0.74 (0.38 to 1.44)	0.37
Vancomycin	31	19.0	1.78 (1.04 to 3.05)	0.36
Penicillin	33	20.2	2.17 (1.28 to 3.66)	0.004
β-lactam and inhibitor	6	3.7	2.37 (0.70 to 8.06)	0.17
First-generation cephalosporin	27	16.6	0.65 (0.40 to 1.07)	0.09
Third-generation cephalosporin	18	11.0	1.21 (0.64 to 2.30)	0.56
Carbapenem	2	1.2	1.05 (0.17 to 6.48)	0.96
Clindamycin	6	3.7	1.35 (0.47 to 3.91)	0.58
Metronidazole	34	20.9	1.70 (1.02 to 2.83)	0.04

^a^ MsSA, methicillin-susceptible *Staphylococcus aureus*; OR, odds ration; CI, confidence interval.

The results for the multivariable models quantifying the specific risk for MSSA associated with levofloxacin or ciprofloxacin after adjustment for other factors are shown in Table 3. In contrast to the findings for MRSA, MSSA case-patients were not significantly more likely to have previously received levofloxacin (OR 0.7; p = 0.3023) and tended to have a smaller risk of having received ciprofloxacin (OR 0.5; p = 0.0571) than the controls.

### Effects of Fluoroquinolone Exposure on MRSA and MSSA

In this study, patients from whom nosocomially acquired MRSA was isolated were approximately three times as likely as those with MSSA to have received prior therapy with levofloxacin or ciprofloxacin (67.6% vs. 22.7%). Adjusting for time at risk, MRSA isolation and prior exposure to both levofloxacin (OR 5.4; p < 0.0001) and ciprofloxacin (OR 2.2; p = 0.0027) were associated. For MSSA, the association was not significant for either levofloxacin (OR 1.1; p = 0.77) or ciprofloxacin (OR 0.74; p = 0.37). After adjusting for multiple risk factors, including exposure to other antimicrobial classes, exposures to levofloxacin (OR 3.4; p < 0.0001) and to somewhat lesser degree ciprofloxacin (OR 2.5; p = 0.0049) were significantly associated with MRSA. For MSSA cases, exposure tended to be protective for ciprofloxacin (OR 0.5; p = 0.0571), but not for levofloxacin (OR 0.7; p = 0.3023) (Figure).

**Figure:**
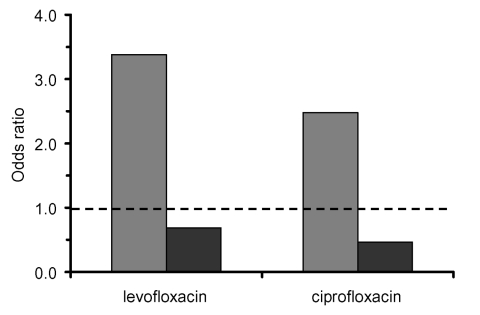
Odds ratios from multivariable analysis for the isolation of MRSA (methicillin-resistant *Staphylococcus aureus*) and MSSA (methicillin-susceptible *Staphylococcus aureus*) after exposure to levofloxacin or ciprofloxacin. Results for MRSA shown in gray and for MSSA in black. All results adjusted for time at risk.

## Discussion

This case-case control study is the first specifically designed to examine the epidemiologic link between fluoroquinolone exposure and the subsequent isolation of *S. aureus* and to specifically differentiate between MRSA and MSSA. After controlling for possible confounders, including exposure to other antimicrobial agents, the results reported here demonstrate a highly significant association between treatment with levofloxacin or ciprofloxacin and subsequent isolation of MRSA, but not MSSA. The magnitude of the risk is less than reported in earlier studies that did not examine this specific question and whose design would tend to bias results.

Substantial variability exists between both case groups and controls with respect to a number of characteristics, including coexisting illnesses, days spent in intensive care, and presence of intravascular catheters. However, such differences, several of which reflect variation in severity of illness and immune status between the groups, do not negatively affect the interpretation of the results. In fact, such dissimilarity, familiar to clinicians, mirrors the very real differences that exist between colonized or infected and uncolonized patients. Because the same control group is employed for both MRSA and MSSA patients, the case-case-control method accounts for any potential confounding attributable to these differences. Moreover, the sustained association between fluoroquinolone exposure and MRSA after adjusting for these surrogates of disease severity and host immune status in the multivariable model further supports the relationship. This conclusion was tested in the handling of patients from the obstetrics service. A priori, we elected to include both cases and controls from the obstetric service. However since this patient population contributed a significantly larger proportion to the control group (18.1%) than to each of the case groups (0.9% and 1.8%), we performed a subgroup analysis to confirm the aforementioned interpretation. When the subgroup analysis was performed excluding patients from the obstetrics service, the overall results of the study were not significantly changed.

Even in the face of intense selection pressure from exposure to antimicrobial drugs, MSSA isolates very rarely develop methicillin resistance. Therefore, any relationship between fluoroquinolones and MRSA probably occurs at the level of host colonization. With this in mind, the findings could be attributed to a number of etiologic mechanisms.

### Competitive Balance between MRSA and MSSA

By eradicating the generally susceptible microorganisms that colonize the skin and mucus membranes (e.g., nares, perirectal area), fluoroquinolone exposure effectively opens an ecologic niche, rendering an inpatient vulnerable to subsequent colonization and infection by the more resistant strains endemic in the hospital, including MRSA. Because fluoroquinolone resistance is relatively rare among strains of MSSA while MRSA isolates tend to be resistant (*23*), the net result could be the replacement of MSSA with MRSA after fluoroquinolone exposure.

### Selection of Preexisting Resistant Subpopulations

Using in vitro analysis, Venezia et al. performed population analysis on fluoroquinolone-susceptible, *mecA*-positive methicillin-heteroresistant strains of *S. aureus*. Growth in the presence of 0.5 MIC of a fluoroquinolone (ciprofloxacin, levofloxacin, moxifloxacin, or gatifloxacin) resulted in a >10-fold increase in the proportion of the population that grew on high concentrations of oxacillin. The increase was directly proportional to the concentration of the fluoroquinolone and could be detected within 8 hours of exposure. The authors conclude that fluoroquinolones might influence oxacillin resistance by selective inhibition or killing more susceptible subpopulations of heteroresistant *S. aureus*. The surviving subpopulations are more resistant to both oxacillin and the quinolones (*24*).

While plausible, neither of the first two explanations is completely supported by the results of our study. Were the relationship between fluoroquinolones and MRSA solely attributable to selective pressure that favors the acquisition or emergence of fluoroquinolone-resistant strains, the same phenomenon would be expected to apply with other antimicrobial agents to which MRSA are also resistant. For example, first-generation cephalosporins have activity against most strains of MSSA but nearly all MRSA isolates are resistant. Nevertheless, first-generation cephalosporins were not identified as a unique risk factor for either organism in this study. The same holds true for exposure to clindamycin.

We think that these results support a third mechanism that is independent of the specific antimicrobial agent activity of the fluoroquinolones. Bisognano et al. have suggested an alternative mechanism by which the fluoroquinolones could uniquely predispose to colonization (and subsequent infection) with *S. aureus*. In a series of in vitro experiments, these researchers have demonstrated that exposure to subinhibitory levels of ciprofloxacin results in increased expression of adherence factors promoting host colonization. Isogenic *S. aureus* mutants expressing varying levels of fluoroquinolone resistance, when grown in the absence of drug, showed little difference in adhesion characteristics when compared with parental strains. However, impressive changes in adhesion were exhibited when strains were grown in the presence of 1/4 MIC of ciprofloxacin. This increased adhesion, which was most pronounced among highly resistant mutants, occurred at therapeutically achievable concentrations of ciprofloxacin and was associated with overexpression of fibronectin-binding protein (*25*). In subsequent work with clinical specimens, the same group showed that 8 of 10 MRSA isolates and 4 of 6 MSSA isolates exhibited increased attachment to fibronectin-coated surfaces after growth in the presence of subinhibitory concentrations of ciprofloxacin. Further, they demonstrated that the effect is mediated at the level of transcription by activation of the *fnb* promoter (*26*). More recently, the same group showed that the SOS RecA-mediated pathway, but not the *agr* or *sar* regulatory elements, plays a role in the control of this phenomenon (*27*,*28*).

While the association between levofloxacin or ciprofloxacin exposure and MRSA colonization could be at least partially explained by the promotion of *S. aureus* adherence, the present epidemiologic study suggests a means by which the phenomenon would apply whether or not the upregulation of fibronectin binding is a feature unique to resistant isolates. Exposure to levofloxacin or ciprofloxacin that promotes increased adherence of both MRSA and MSSA would have the net effect of increasing the likelihood of recovery of fluoroquinolone-resistant isolates. Because most MSSA isolates, as opposed to MRSA, are susceptible to the fluoroquinolones (92% vs. 3% in this study), MSSA would be selectively eliminated by the antimicrobial agent action of either drug before colonization could be established. In summary, fluoroquinolone exposure would have the dual effect of promoting *S. aureus* colonization while selectively eradicating MSSA strains; the net effect of which is to favor MRSA colonization.

In addition to the overall effect of the fluoroquinolones, we also sought to examine the difference in risk associated with ciprofloxacin versus levofloxacin. Although we observed a trend toward greater risk for MRSA after exposure to levofloxacin than ciprofloxacin, the difference was not significant. Such a difference, if confirmed by further investigation, would be unexpected. On the basis of comparative MIC data, levofloxacin is considered more active than ciprofloxacin against susceptible isolates of *S. aureus*. Our results indicate that levofloxacin may be more likely to promote MRSA and suggest that the effect of levofloxacin in promoting colonization may be stronger than that of ciprofloxacin. Any differences between fluoroquinolones, if proven, would have important implications regarding the clinical decision to choose a particular fluoroquinolone and could shed light on the mechanism of the relationship between these agents and MRSA.

The association between fluoroquinolone exposure and MRSA, established here using rigorous epidemiologic methods, serves as a reminder that the risk factors associated with emerging antimicrobial resistance may not always be predictable or intuitively obvious. Careful consideration must be given to the clinical implications of these findings. In the case of fluoroquinolones and MRSA, decisions promoting the use of a single antimicrobial drug or class of agents could have unforeseen effects on the emergence of antimicrobial resistance.
